# Diagnostic accuracy of LAMP assay for HBV infection

**DOI:** 10.1002/jcla.23281

**Published:** 2020-03-10

**Authors:** Chu‐Mao Chen, Shi Ouyang, Li‐Ying Lin, Li‐Juan Wu, Tian‐Ao Xie, Juan‐Jiang Chen, Zhen‐Xing Li, Guo‐Dong Zhu, Tian‐Xing Ji, Zhi‐Yong Pan, Yong Xia, Xu‐Guang Guo

**Affiliations:** ^1^ Department of Clinical Laboratory Medicine The Third Affiliated Hospital of Guangzhou Medical University Guangzhou China; ^2^ Department of Infectious Disease The Fifth Affiliated Hospital of Guangzhou Medical University Guangzhou China; ^3^ Department of Clinical Medicine The Third Clinical School of Guangzhou Medical University Guangzhou China; ^4^ Baoan Maternal and Child Health Hospital Jinan University Shenzhen China; ^5^ Department of Respiratory Medicine The Third Affiliated Hospital of Guangzhou Medical University Guangzhou China; ^6^ Department of Geriatrics Guangzhou First People's Hospital School of Medicine South China University of Technology Guangzhou China; ^7^ Department of Clinical Medicine The Second Affiliated Hospital of Guangzhou Medical University Guangzhou China; ^8^ Key Laboratory for Major Obstetric Diseases of Guangdong Province The Third Affiliated Hospital of Guangzhou Medical University Guangzhou China; ^9^ Key Laboratory of Reproduction and Genetics of Guangdong Higher Education Institutes The Third Affiliated Hospital of Guangzhou Medical University Guangzhou China

**Keywords:** HBV, LAMP, PCR, rapid diagnosis accuracy, systematic evaluation

## Abstract

**Background:**

Detection of hepatitis B virus (HBV) is vital for the diagnosis of hepatitis B infection. A novel test loop‐mediated isothermal amplification (LAMP) has been successfully applied to detect various pathogens. However, the accuracy of LAMP in diagnosing HBV remains unclear. Therefore, in the present study, the accuracy of LAMP for HBV detection was evaluated systematically.

**Methods:**

Embase, Cochrane Library, and PubMed databases were searched for studies using LAMP to detect HBV. Then, two researchers extracted data and assessed the quality of literature using the QUADAS‐2 tool independently. I^2^ statistic and chi‐square test were analyzed to investigate the heterogeneity, and Deek's funnel plot assessed the publication bias. The pooled sensitivity (SEN), specificity (SPE), positive LR (PLR), negative LR (NLR), diagnostic odds ratio (DOR), and 95% confidence intervals were displayed in forest plots. We calculated the area under the curve (AUC) to assess the overall efficiency of LAMP for HBV detection.

**Results:**

A total of nine studies with 1298 samples were finally included in this evaluation. The pooled sensitivity and specificity of HBV detection were 0.91 (95% CI: 0.89 ~ 0.92) and 0.97 (95% CI: 0.94 ~ 0.99), respectively. The PLR, NLR, and DOR were 16.93 (95% CI: 6.15 ~ 46.55), 0.08 (95% CI: 0.05 ~ 0.14), and 397.57 (95% CI: 145.41 ~ 1087.07). Besides, the AUC was 0.9872, and Deek's plot suggested that there existed publication bias in the studies.

**Conclusion:**

Compared with PCR, LAMP is a simple, rapid, and effective assay to diagnose HBV. However, additional evidence is essential to confirm that LAMP can replace other methods in diagnosing HBV infection.

## 1 INTRODUCTION

According to the report of the World Health Organization (WHO) in 2015, the hepatitis B virus (HBV) infected 275 million people worldwide and presented a threat to global health. The number of deaths caused by hepatitis B was 887 000. A total of 1.1 million new infections were detected in 2017. The region with the highest prevalence of hepatitis B was in the Western Pacific and Africa, with adult infection rates 6.2% and 6.1%, respectively.^1^, ^2^ HBV is a circular, 3.2 kbp partially double‐stranded DNA with eight genotypes (A–H).^3^, ^4^ The transmission of the virus occurs mainly through blood or mother‐to‐child.^5^, ^6^ HBV is one of the leading causes of acute and chronic hepatitis and liver cancer, which is often co‐infected with the hepatitis C virus and human immunodeficiency virus.^7^, ^8^ And the gene expression of TLR1, TLR6, and NEAT1 may attribute to the innate immune response to enhance the chronic infection.^9^ Some studies have determined that the HBV level was associated with HBeAg.^6^ Therefore, a highly accurate method for the detection of HBV is essential for the early diagnosis, treatment, and prognosis.

Various techniques, such as enzyme‐linked immunosorbent assay (ELISA) and quantitative PCR (qPCR), have been widely used to detect the hepatitis B surface antigen (HBsAg) and HBV‐DNA.^10^, ^11^, ^12^, ^13^, ^14^ Jean et al (2018) determined the sensitivity of BIOSYNEX IMMUNOQUICK^®^ RDT for HBsAg detection was 78%, higher than the previous report.^5^ Matsuo et al (2017) applied “Lumipulse HBsAg‐HQ” to detect the HbsAg and its variants, finding that HBsAg‐HQ assay had high sensitivity and lower LOD to detect HBV.^15^ However, the sensitivity of the immunization method was not high in screening hepatitis B of early stage, while the sensitivity of the PCR technique to detect HBV‐DNA of acute infection was higher.^16^, ^17^ PCR‐based detection has certain advantages in sensitivity, pollution control, and virus quantity test. Nevertheless, these time‐consuming and expensive methods require high personnel and equipment. Thus, an alternative approach, loop‐mediated isothermal amplification (LAMP), has been under intensive investigation.

LAMP was developed by Notomi et al (2000)^18^ and successfully applied for the detection of HBV and other pathogenic microorganisms.^19^, ^20^, ^21^, ^22^ It has high accuracy for the detection of microorganisms. For example, the LAMP can detect NDM‐1 gene with high specificity in 45 minutes.^23^ However, LAMP, combined with other technologies, can improve the efficiency of diagnosis. To identify the viable bacteria, Wu et al (2017)^24^ applied an RT‐LAMP assay to detect the 16S rRNA of Mycobacterium tuberculosis and found that RT‐LAMP was 10‐fold higher in sensitivity than that of the LAMP. Four specific primers are designed to amplify the six particular regions of the target sequence. The Bst DNA polymerase is used for strand displacement‐DNA synthesis. LAMP can be performed at about 65°C in a water bath for 60 minutes. The naked eyes or amplification curve observed the reaction.

In recent years, the detection of HBV by LAMP has been established, but the overall evaluation has not been studied thoroughly. Therefore, we combined the previous research data and made a systematic analysis to reveal the accuracy of LAMP in detecting HBV.

## 2 MATERIALS AND METHODS

### 2.1 Electronic searches

We conducted this systematic evaluation according to the standard guidelines.^25^, ^26^, ^27^, ^28^ The search using databases such as PubMed, Embase, and Cochrane Library retrieved relevant studies published from January 1, 2000, to date without language restriction. The search words (((Hepatitis) OR (Hepatitis B) OR (HBV) OR (Hepatitis B virus)) AND ((Loop‐mediated isothermal amplification) OR (LAMP))) were used to identify the studies evaluating LAMP accuracy in HBV infection. We retrieved all the studies searched on the databases and checked their references manually for eligible studies.

### 2.2 Study screening and selection

Two investigators (Chu‐Mao Chen and Li‐Juan Wu) independently checked the title and abstract to screen out the relevant literature; any discrepancies were settled by a third investigator (Tian‐Ao Xie). The studies that met the eligibility criteria were included in this evaluation.

### 2.3 Inclusion and exclusion criteria

We analyzed and included the literature that fulfilled the following criteria: (a) the purpose was to evaluate the LAMP in the diagnosis of HBV; (b) comparison between the index test and the gold‐standard method; and (c) the studies provided data such as true positive (TP), false positive (FP), true negative (TN), and false negative (FN). However, the studies were excluded if (a) the data provided could not calculate the sensitivity and specificity; (b) the gold‐standard method was not precise or not used; (c) the number of cases was <10; and (d) repeated published studies.

### 2.4 Data extraction

The necessary information was extracted independently by two investigators from each of the included studies, such as the year of publication, reference method, and the number of samples. Additionally, the diagnostic characteristics of LAMP, such as TP, FP, TN, and FN, were extracted. Then, we reviewed the information obtained by two investigators, and the disagreement was settled by a third investigator.

### 2.5 Quality assessment

Two investigators assessed the quality of the included studies independently based on the QUADAS‐2 standard.^29^ The quality of the included studies was evaluated by two investigators. The QUADAS‐2 comprised of eleven criteria in four domains (patient selection, index test, gold‐standard method, and flow and timing). We used “Yes,” “Unclear,” and “No” to assess the risk of bias of the studies. The first three assessments considered the clinical applicability of the method.

### 2.6 Statistical analysis

Statistical analysis and data synthesis were performed according to the standard method using the Meta‐Disc 1.4 software.^30^

### 2.7 Publication bias

In a systematic evaluation, we tested the publication bias of the included studies by drawing Deek's funnel plot using Stata 12.0 software.

### 2.8 Threshold effect detection

Spearman's correlation coefficient was calculated to explore the threshold effect of the included studies. A negative correlation between the sensitivity and specificity suggested a threshold effect or vice versa.^31^

### 2.9 Investigations of heterogeneity

We performed chi‐square test and I^2^ statistic to assess the heterogeneity among the included studies.^32^ When a heterogeneity effect was detected, the random‐effects model was selected. If the heterogeneity was small or absent, the fixed‐effects model was implemented.

### 2.10 Data synthesis

We used the Meta‐Disc 1.4 software to merge the sensitivity, specificity, positive LR (PLR), and negative LR (NLR) using an appropriate model. The results were shown in forest plots. Also, SROC was applied to calculate the AUC. Then, DOR and AUC were used to evaluate the overall efficiency of the LAMP assay. Each item would combine the 95% confidence interval (95% CI).^33^

## 3 RESULTS

### 3.1 Literature search

**Figure 1:**
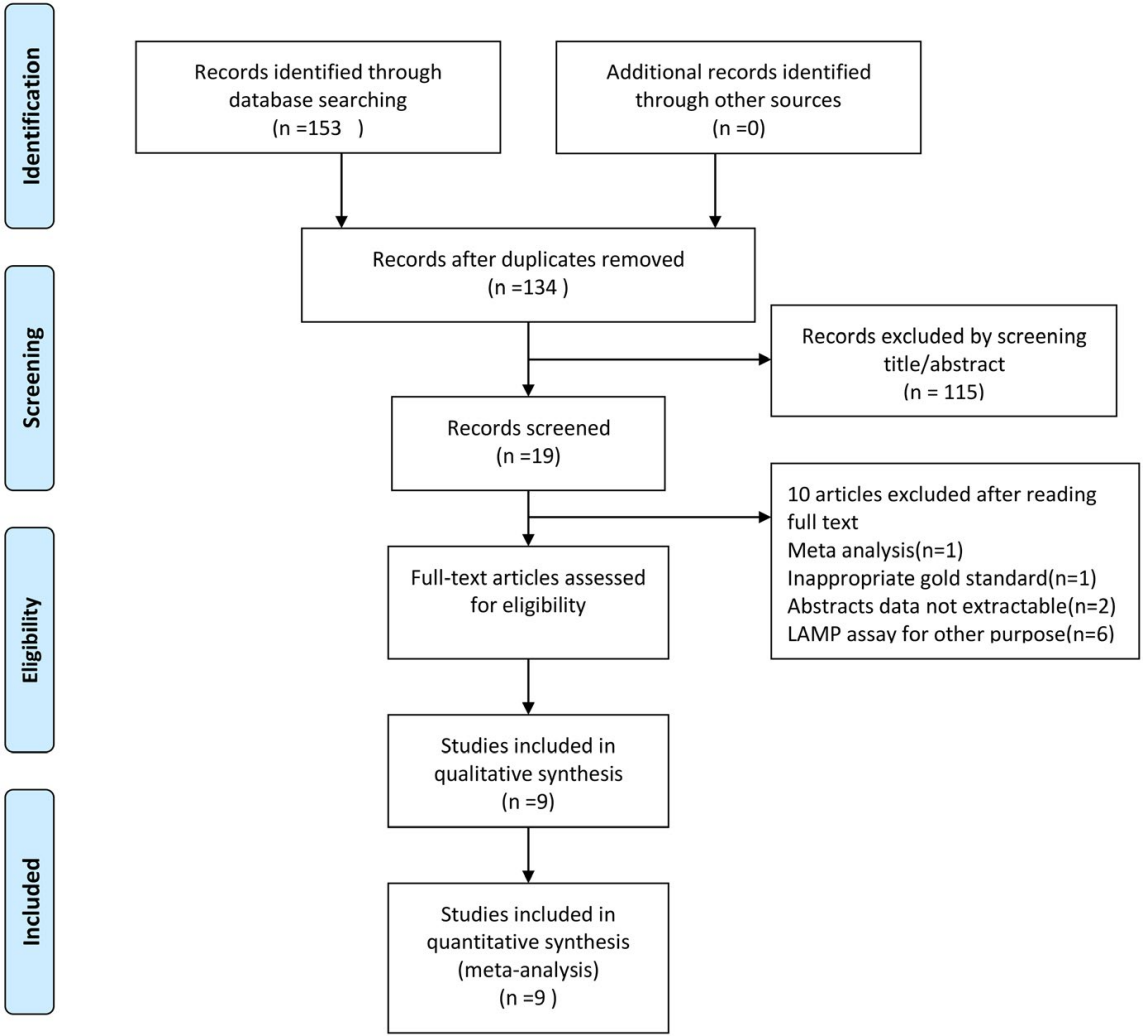
Flow diagram of study identification and inclusion

Based on the current search strategy, we identified 153 related articles: 103 articles were from PubMed, 25 from Embase, and 25 from the Cochrane Library. After excluding 19 repetitive items, we screened the titles, abstracts, and full text. Finally, 125 ineligible articles were excluded according to the criteria, and nine articles were included.^19^, ^34^, ^35^, ^36^, ^37^, ^38^, ^39^, ^40^, ^41^ The process is represented in Figure 1.

### 3.2 Study characteristics

The characteristic details of the articles are shown in Table 1. In this study, a total number of 1298 samples in the studies published from 2000 to 2019 were analyzed, including 327 serum samples, 539 plasma samples, and 432 blood samples from five countries. In the reference methods, three articles compared LAMP to traditional PCR, while four compared it to real‐time PCR. Moslemi et al adopted COBAS PCR, while Nyan et al used the Procleix Ultrio Plus assay. The incubation temperatures of the experiments were similar at approximately 65 ℃. Of the included articles, two were from the United States, two from Iran, three from China, and the remaining from India and Vietnam, respectively.

**Table 1:** Details of the included studies

Author	Year	Country	Reference method	Detection Method	Target Gene	Incubation Time (min)	Incubation Temperature (℃)	Sample type	Sample size	TP	FP	FN	TN
Chen^41^	2019	America	PCR	LAMP	Pre‐Surface/Surface antigen region	60	60	Plasma	138	107	0	20	11
Quoc^39^	2018	Vietnam	Rt‐ PCR	LAMP	Not mentioned	30	63	Blood	30	19	0	0	11
Zhao^35^	2016	China	FQ‐PCR	LAMP	S gene	20	65	Serum	75	60	0	5	10
Nyan^34^	2014	America	Procleix Ultrio Plus assay	LAMP	S gene; part of overlap polymerase region of HBV	60	60	Plasma	182	69	6	0	107
Joshi^36^	2013	India	Rt‐ PCR	LAMP	Precore and e antigen region	Unclear	Unclear	Plasma	19	13	2	0	4
A.Iadi^38^	2012	Iran	PCR	LAMP	Common sero and genotype (accession No. Ay771794)	60	66	Plasma	200	172	0	16	12
Cai,Z^40^	2011	China	Rt‐PCR	LAMP	S region	60	60	Serum	141	105	0	6	30
Moslemi^37^	2009	Iran	COBAS‐PCR	LAMP	HBs region	60	66	Serum	111	101	0	3	7
Cai,T^19^	2008	China	PCR	RtF‐LAMP	Pre‐core/core region	60	63	Blood	402	295	0	47	60

Abbreviations: FN, false negative; FP, false positive; FQ‐PCR, fluorescence quantitative PCR; RtF‐LAMP, real‐time fluorescence LAMP; Rt‐PCR, real‐time PCR; TN, true negative; TP, true positive.

### 3.3 Quality assessment of the study

**Figure 2:**
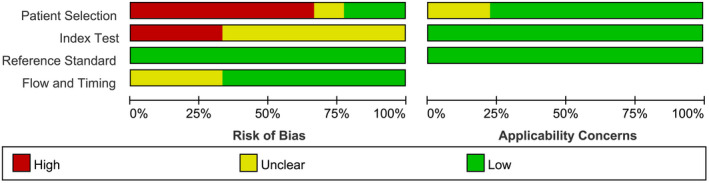
Risk of bias and applicability concerns graph

**Figure 3:**
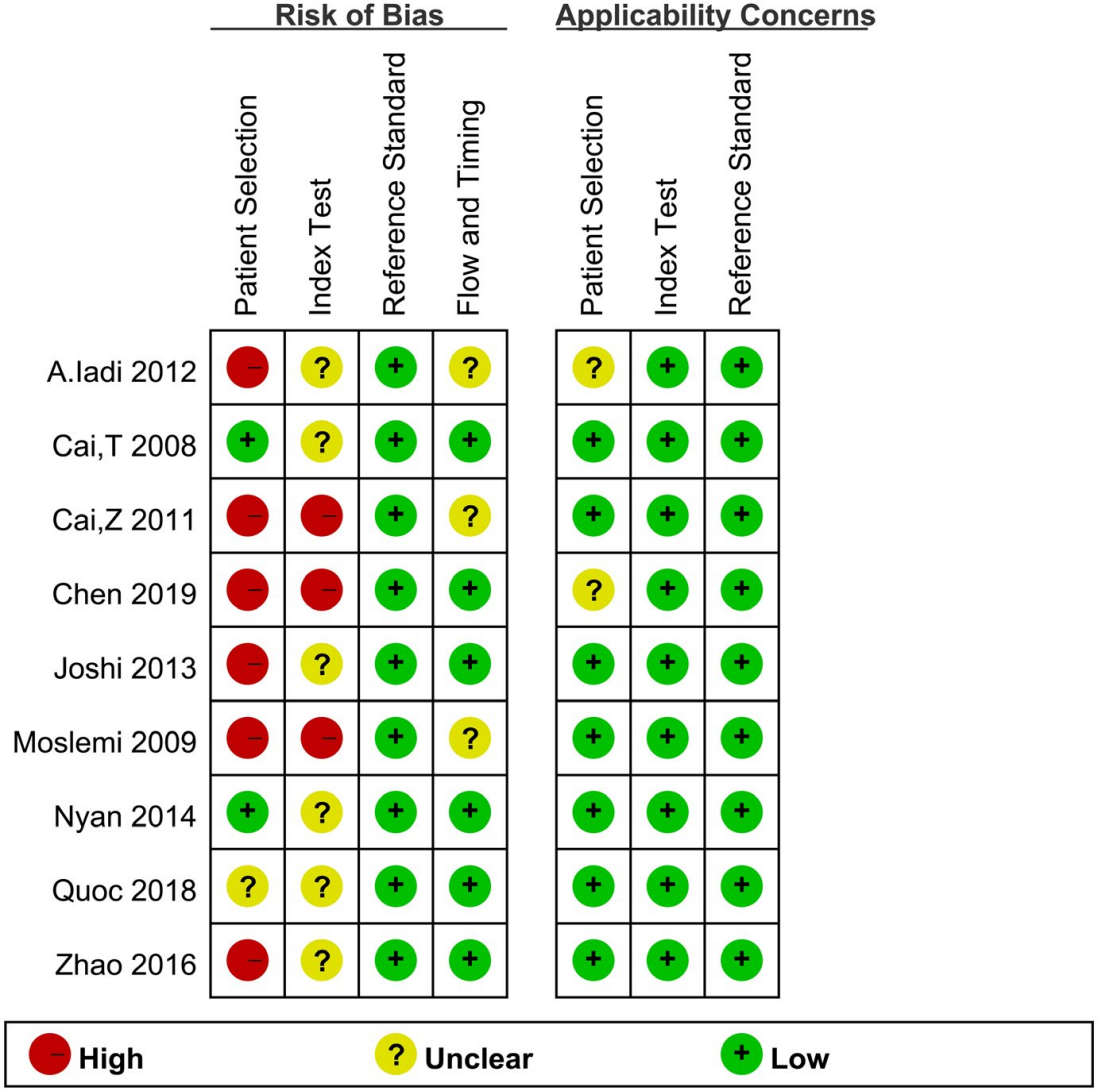
Risk of bias and applicability concerns summary

According to QUADAS‐2,^29^ the methodological quality of nine articles was evaluated independently by two researchers. The criteria we used to assess the methodological quality were catalogued in Table S1. Quality assessment of the included studies was shown in Table S2. The risk of bias and applicability results are presented in Figures 2 and 3 and Table S3. In terms of patient selection, six studies were evaluated to offer a high risk of bias because they included the HBV‐confirmed cases or set up a case‐control trial, or did not avoid inappropriate exclusion, while two studies were evaluated as low risk of bias. Nevertheless, the remaining studies were not clear because the description of the case was inadequate. In the aspect of the index test, approximately 33% of the studies were considered as low risk of bias while the remaining were at unclear risk due to their indistinct thresholds, or the results of the gold‐standard method were known when explaining the index test.

Furthermore, concerning the reference standard, all the included studies were judged to be at low risk of bias. The results of the index test were unknown while explaining the results of the gold‐standard method. Regarding the flow and timing domain, about 67% of the studies were rated as low risk of bias. The rest were at unclear risk due to the lack of descriptions about the appropriate time interval between the index test and the gold‐standard method. In terms of the applicability of case selection, that of two studies was not clear, and the remaining were at low risk.

### 3.4 Threshold effect

Spearman's correlation coefficient was 0.561, and *P*‐value = .116 suggested that no threshold effect could not cause heterogeneity among the included studies.

### 3.5 Diagnostic accuracy

**Figure 4:**
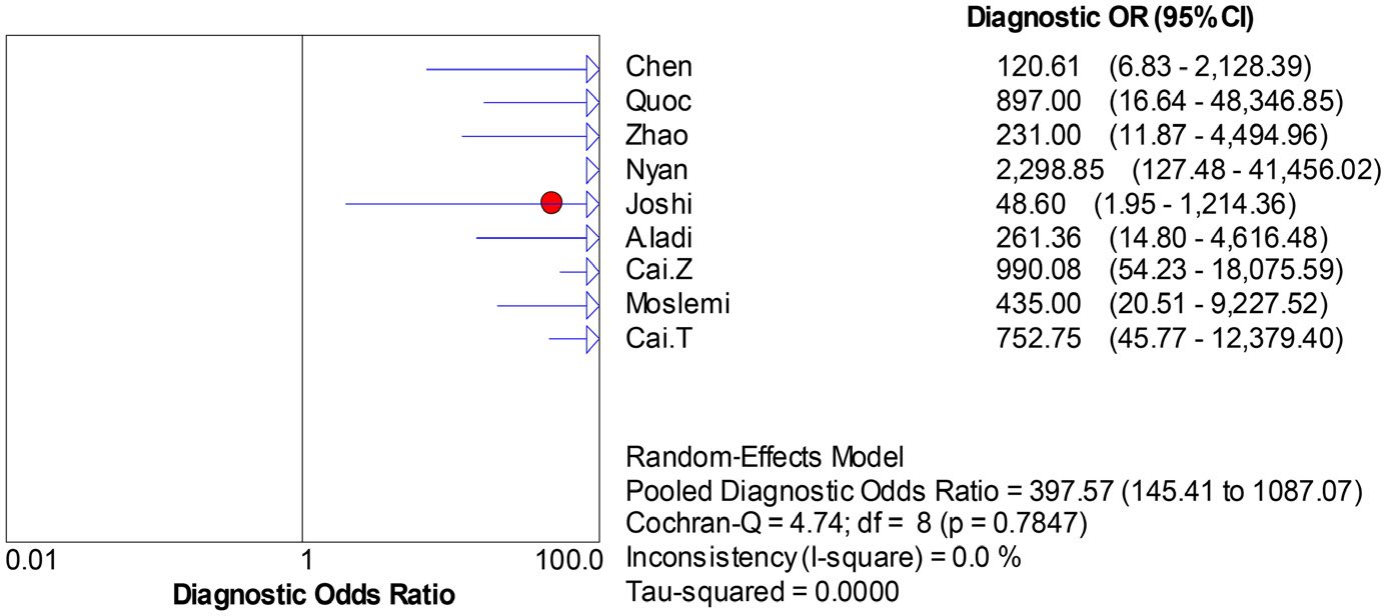
Forest plots for the pooled diagnostic OR of LAMP

**Figure 5:**
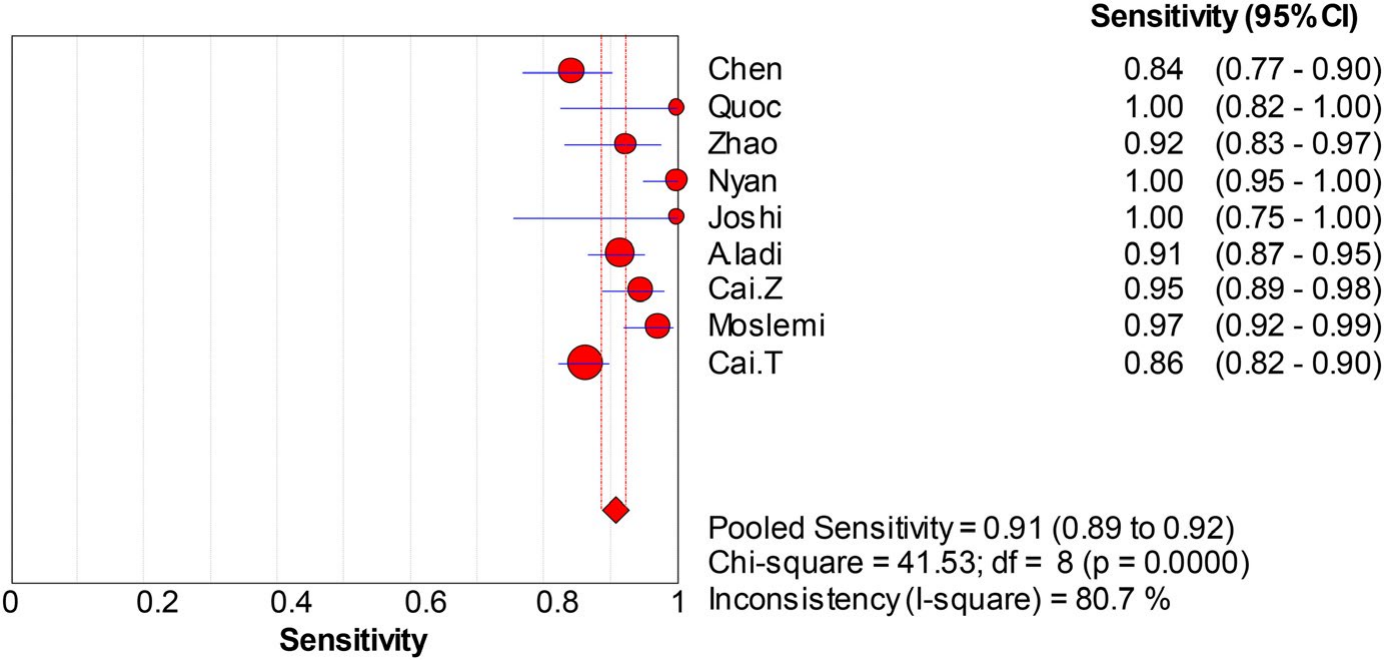
Forest plots for the pooled sensitivity of LAMP

**Figure 6:**
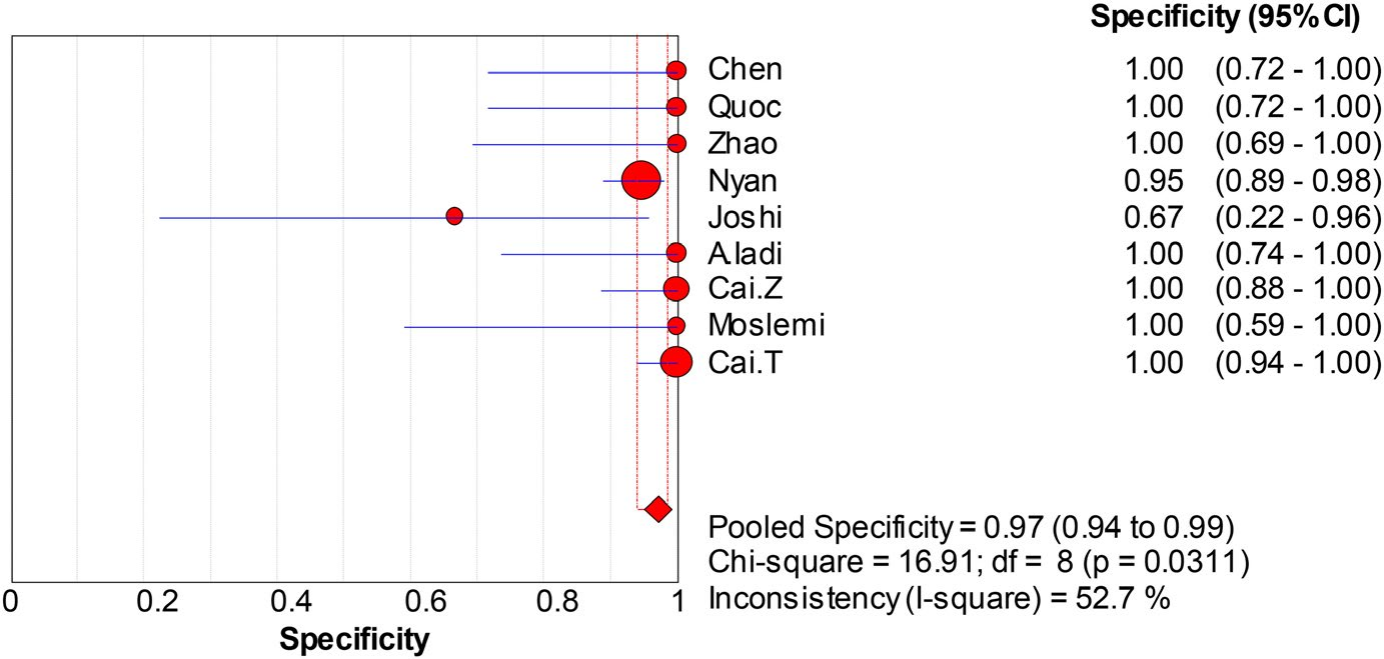
Forest plots for the pooled specificity of LAMP

**Figure 7:**
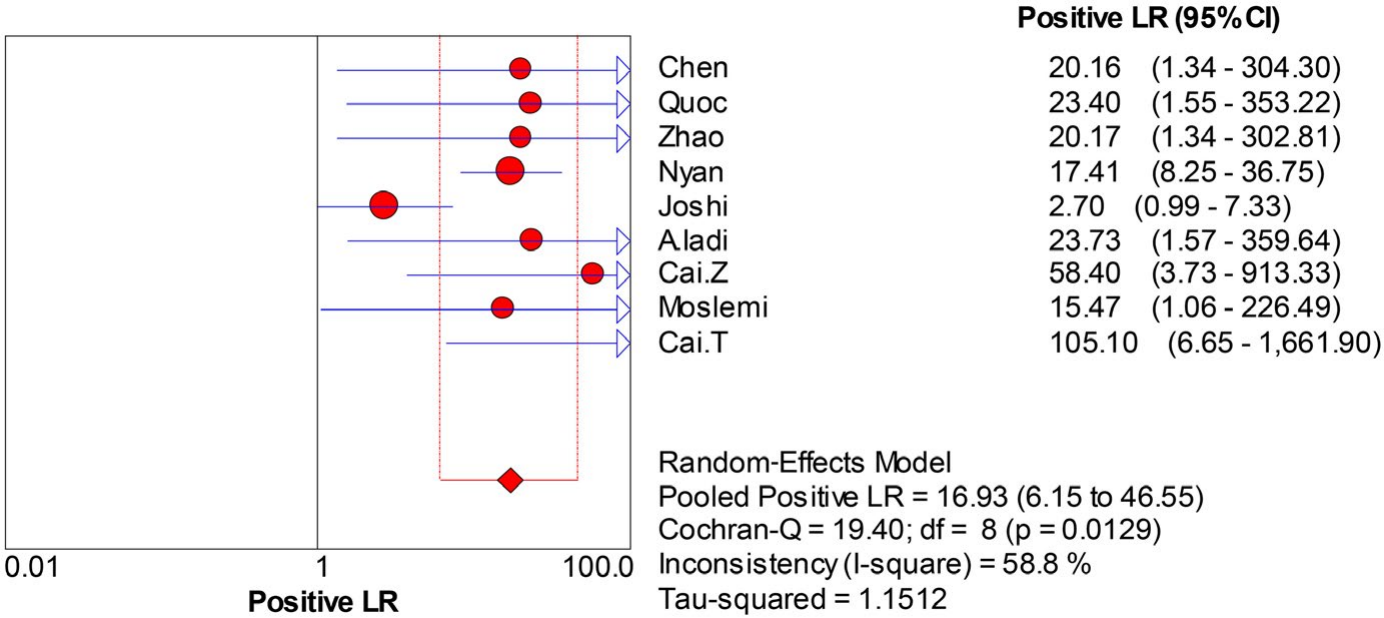
Forest plots for the pooled positive LR of LAMP

**Figure 8:**
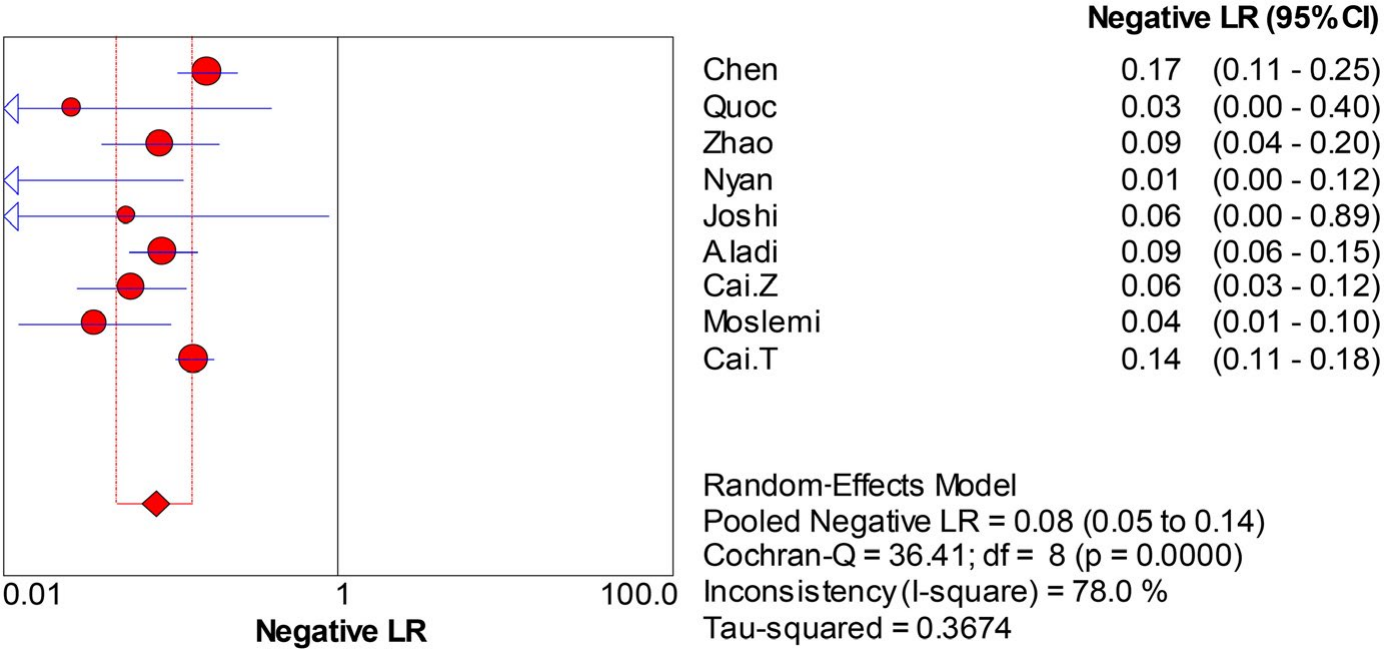
Forest plots for the pooled negative LR of LAMP

**Figure 9:**
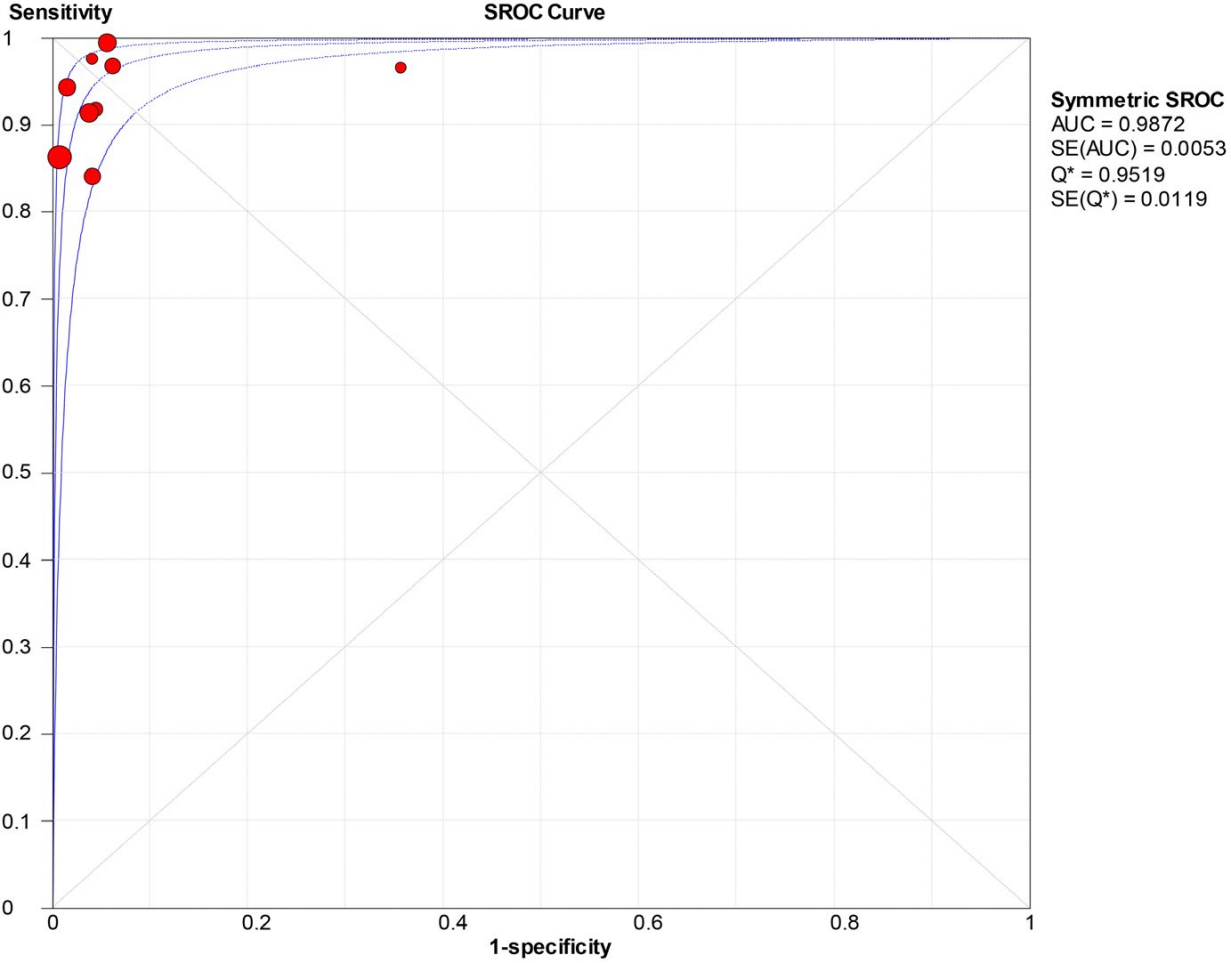
SROC of HBV infections detected by LAMP

The forest plots were drawn with the random‐effects model using the Meta‐Disc 1.4 software. As presented in Figure 4, we obtained the following results: Cochran‐Q = 4.74, *P* = .7847, and inconsistency = 0.0%, which indicated that the threshold effect caused no heterogeneity. Then, we merged the data to evaluate the diagnostic accuracy of LAMP (Figures 5, 6, 7, 8). The combined sensitivity, specificity, PLR, and NLR were 0.91 (95% CI: 0.89‐0.92; I^2^: 80.7%), 0.97 (95% CI: 0.94‐0.99; I^2^: 52.7%), 16.93 (95% CI: 6.15‐46.55; I^2^: 58.8%), and 0.008 (95% CI: 0.05‐0.14; I^2^: 78.0%), respectively. The Cochran‐Q value of SEN, SPE, PLR, and NLR was 41.53, 16.91, 19.40, and 36.41, respectively. However, the *P*‐values of the chi‐square test in each study were <.05, suggesting heterogeneity among the studies. As shown in Figure 4, the DOR was 397.57 (95% CI: 145.41‐1087.07). Thus, we adopted a random‐effects model to draw the SROC (Figure 9). The results showed that the AUC = 0.9872 and Q index was 0.9519 (SE = 0.0119). Both DOR and SROC suggested that LAMP had high diagnostic accuracy for the HBV infection.

### 3.6 Publication bias

**Figure 10:**
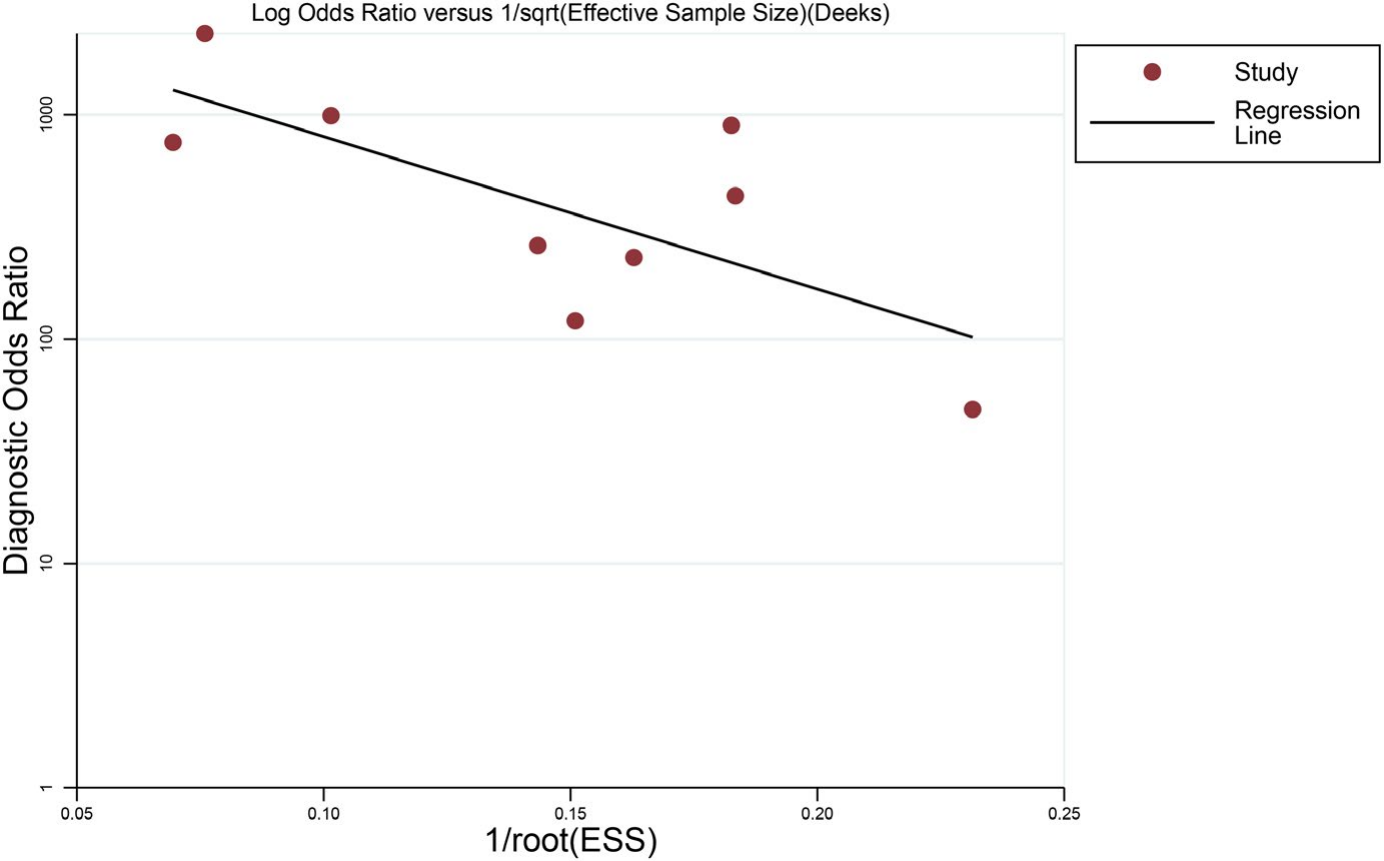
Deeks’ funnel plot asymmetry test to assess publication bias

Deek's funnel plot (Figure 10) was drawn using Stata 12.0. The test indicated a potential publication bias in the included studies (*P* = .026).

## 4 DISCUSSION

LAMP is a novel nucleic acid amplification method, which amplifies six regions on the target sequence using four specific primers and Bst DNA polymerase at 65°C. Currently, several pathogenic bacteria and viruses are detected by LAMP; however, only a few studies used LAMP technology to detect HBV. In the present study, we searched the relevant databases and established strict screening criteria. Finally, nine studies were included. Two researchers independently extracted the data, and the accuracy of LAMP in detecting HBV was evaluated.

The results of this analysis showed high sensitivity (0.91) and specificity (0.97) of LAMP in the diagnosis of HBV. The specificity increased as compared to the study by Zhou et al (SEN = 0.92, SPE = 0.86),^42^ suggesting that the LAMP improved the differentiation between HBV and non‐HBV infection. Moreover, the combined PLR and NLR were 16.91 and 0.08, respectively. PLR > 10 and NLR < 0.1 indicated that LAMP had a low missed diagnosis and misdiagnosis rate in HBV detection. The area under the SROC curve (AUC), Q index, and DOR were used to assess the overall performance of the diagnostic tests^43^. In this study, the combined DOR was 397.57, AUC was 0.9872, and the Q index was 0.9519, higher than that described previously (Zhou et al 2017) (DOR = 311.09, AUC = 0.986, Q = 0.949), which showed that LAMP has high accuracy in detection of HBV.

Further analysis revealed publication bias among the included studies (*P* = .026). However, the value of the I^2^ statistic of pooled SEN, SPE, PLR, and NLR was >50%, and the *P*‐value of the chi‐square test was <.05, which indicated the presence of heterogeneity in the studies. Thus, the combined results should be interpreted with caution. The threshold effect is one of the sources of heterogeneity. Spearman's correlation coefficient in the current study was 0.561 and *P* = .116, suggesting the lack of threshold effect in the included studies. Then, the sample type, condition, experimental design, and protocol were assessed carefully in each of the studies. We found two researches that performed a case‐control trial to improve the accuracy of diagnosis.^35^, ^36^ In the study by Izadi,^38^ the samples were stored for a prolonged period, leading to a false‐negative result with the reference method. However, the MgP_2_O_3_ would increase with the synthesis of DNA. High turbidity caused by MgP_2_O_3_ exerted a specific effect on the observation of the results.^38^ Besides, the selection and success of the target gene amplification are essential to the LAMP reaction. Nevertheless, whether the target sequences are consistent with other studies is not yet clarified from these two studies.^34^, ^39^ The factors such as sample size, viral load, the severity of illness, and experimental operators in these studies would cause the heterogeneity.

Nevertheless, the present systematic evaluation has several limitations. First, no grouping analysis was carried out due to the limited number of published articles, the small size of samples, and the lack of comparability. Therefore, we need to improve the evaluation after the increase in the number of clinical samples. Second, the majority of the studies included well‐diagnosed or healthy cases, overestimating the diagnostic accuracy of the LAMP method. Third, the existence of general quality research led to a certain degree of heterogeneity. Finally, the detection ability of the reference method was not stronger than that of LAMP technology, which led to false‐positive results, thereby underestimating the specificity of the LAMP method.

In conclusion, LAMP is a rapid, sensitive, and specific detection method, providing reliable experimental results for the diagnosis of HBV infection. Also, it improves the treatment of patients and reduces the financial burden. With the development and maturity of technology, LAMP might become a primary auxiliary diagnostic tool for HBV infection.

## CONFLICT OF INTEREST

The authors declare that there are no competing interests associated with the manuscript.

## AUTHOR'S CONTRIBUTION

CM Chen, LY Lin, TA Xie, and XG Guo conceived and designed the experiments. CM Chen, JJ Chen, ZX Li, and S Ouyang analyzed the studies and extracted the data available. LJ Wu, TA Xie, LY Lin, and GD Zhu contributed to making the tables. CM Chen, TA Xie, and ZY Pan contributed to the publication of figures. All members participated in the writing, review, discussion, and revision of the manuscript and adopted the final version unanimously.

## Supporting information

Table S1

Table S2

Table S3
