# Supplementary material for: Diagnostic accuracy of LAMP assay for HBV infection
Source: J Clin Lab Anal. 2020 Mar 10;34(7):e23281. doi: 10.1002/jcla.23281 (PMC7370716; doi:10.1002/jcla.23281)
Supplement: Supplementary file 1 — Table S1 [file JCLA-34-e23281-s001.rtf]

Table S1  Quality assessment tool for diagnostic accuracy studies
DOMAIN 1: PATIENT SELECTION	
Was a consecutive or random sample of patients enrolled? 	Yes/No/Unclear 	
Was a case-control design avoided?	Yes/No/Unclear 	
Did the study avoid inappropriate exclusions?	Yes/No/Unclear 	
Could the selection of patients have introduced bias?	RISK: LOW/HIGH/UNCLEAR 	
DOMAIN 2: INDEX TEST(S)	
Were the index test results interpreted without knowledge of the results of the reference standard? 	
Yes/No/Unclear 	
If a threshold was used, was it pre-specified? 	Yes/No/Unclear 	
Could the conduct or interpretation of the index test have introduced bias? 	RISK: LOW /HIGH/UNCLEAR 	
DOMAIN 3: REFERENCE STANDARD	
Is the reference standard likely to correctly classify the target condition?	
Yes/No/Unclear	
Were the reference standard results interpreted without knowledge of the results of the index test? 	
Yes/No/Unclear	
Could the reference standard, its conduct, or its interpretation have introduced bias? 	
RISK: LOW /HIGH/UNCLEAR 	
DOMAIN 4: FLOW AND TIMING	
Was there an appropriate interval between index test(s) and reference standard?	
Yes/No/Unclear	
Did all patients receive a reference standard? 	Yes/No/Unclear	
Did patients receive the same reference standard? 	Yes/No/Unclear	
Were all patients included in the analysis?	Yes/No/Unclear	
Could the patient flow have introduced bias? 	RISK: LOW /HIGH/UNCLEAR 	
