# Supplementary material for: Diagnostic accuracy of LAMP assay for HBV infection
Source: J Clin Lab Anal. 2020 Mar 10;34(7):e23281. doi: 10.1002/jcla.23281 (PMC7370716; doi:10.1002/jcla.23281)
Supplement: Supplementary file 2 — Table S2 [file JCLA-34-e23281-s002.rtf]

Author	Year	QUADAS-2	
		1	2	3	4	5	6	7	8	9	10	11	
Chen	2019	N	UC	N	N	UC	Y	Y	Y	Y	Y	Y	
Quoc	2018	Y	UC	Y	Y	UC	Y	Y	Y	Y	Y	Y	
Zhao	2016	UC	N	N	UC	Y	Y	Y	Y	Y	Y	Y	
Nyan	2014	Y	Y	Y	Y	UC	Y	Y	Y	Y	Y	Y	
Joshi	2013	N	N	N	UC	Y	Y	Y	Y	Y	Y	Y	
A.Iadi	2012	N	UC	UC	Y	UC	Y	Y	UC	UC	UC	Y	
Cai,Z	2011	N	UC	UC	N	UC	Y	Y	UC	Y	Y	Y	
Moslemi	2009	N	UC	UC	N	UC	Y	Y	UC	Y	Y	Y	
Cai,T	2008	Y	Y	Y	Y	UC	Y	Y	Y	Y	Y	Y	
Table S2  Quality assessment of the inclued studies.
Abbreviations:  Y, Yes;  N, No;  UC, Unclear
