# Supplementary material for: Diagnostic accuracy of LAMP assay for HBV infection
Source: J Clin Lab Anal. 2020 Mar 10;34(7):e23281. doi: 10.1002/jcla.23281 (PMC7370716; doi:10.1002/jcla.23281)
Supplement: Supplementary file 3 — Table S3 [file JCLA-34-e23281-s003.rtf]

       Table S3  QUADAS-2 assessment of methodologic quality of the inclued studies.
Author	Year	Risk of bias		Applicability Concerns	
		Patient selection	Index test	Reference standard	Flow and timing		Patient selection	Index test	Reference standard	
Chen	2019	HR	HR	LR	LR		UC	LR	LR	
Quoc	2018	UC	UC	LR	LR		LR	LR	LR	
Zhao	2016	HR	UC	LR	LR		LR	LR	LR	
Nyan	2014	LR	UC	LR	LR		LR	LR	LR	
Joshi	2013	HR	UC	LR	LR		LR	LR	LR	
A.Iadi	2012	HR	UC	LR	UC		UC	LR	LR	
Cai,Z	2011	HR	HR	LR	UC		LR	LR	LR	
Moslemi	2009	HR	HR	LR	UC		LR	LR	LR	
Cai,T	2008	LR	UC	LR	LR		LR	LR	LR	
Abbreviations: LR, low risk;  HR, high risk;  UC, unclear risk
